# Internal Cerebral Vein in Susceptibility-Weighted Imaging: A Reliable Tool to Differentiate Among Calcification, Microbleed, and Gross Hemorrhage in Brain Tumors

**DOI:** 10.7759/cureus.61166

**Published:** 2024-05-27

**Authors:** Minth Punpichet, Chalisa Limcharoenchai, Kamolkan Suthiwartnaruput, Theeraphol Panyaping

**Affiliations:** 1 Department of Radiology, Phramongkutklao Hospital and College of Medicine, Bangkok, THA; 2 Department of Radiology, Phramongkutklao Hospital, Bangkok, THA; 3 Department of Diagnostic and Therapeutic Radiology, Faculty of Medicine, Ramathibodi Hospital, Mahidol University, Bangkok, THA

**Keywords:** swi, gross hemorrhage, microbleed, calcification, brain tumor, internal cerebral vein

## Abstract

Background and objective

Susceptibility-weighted imaging (SWI) sequence is crucial for brain MRI examinations, as it is equipped with a high sensitivity to detect calcification, microbleed, and gross hemorrhage. Intracranial venous structures such as the superior sagittal sinus (SSS) and cortical veins are used as reference structures in phase image SWI to differentiate diamagnetic and paramagnetic substances. Our study focuses on the internal cerebral vein (ICV) as another reliable reference structure. We aimed to analyze the diagnostic accuracy and detectability of calcification and hemorrhagic components in brain tumors using ICV, cortical veins, and SSS as references on phase image SWI, with CT scans for comparison.

Material and methods

A retrospective review of calcification and hemorrhagic components in brain tumors was conducted using MRI and CT from January 2017 to June 2023.

Results

The study included a total of 192 patients with brain tumors. For calcification components (63 cases), ICV and cortical veins as reference structures showed excellent sensitivity (96.8%), specificity (100%), and accuracy (98.9%). SSS demonstrated slightly lower detectability but maintained high sensitivity (96.5%), specificity (100%), and accuracy (98.8%) levels. No statistical differences were noted among these reference structures (p>0.05) and excellent interobserver agreement (Cohen's Kappa of 1) was observed.

Conclusions

The ICV is located in the central image, is large, without any nearby arteries, and is easy to identify using SWI phase images. Using the ICV as a reference to characterize intratumoral calcification, microbleed, and hemorrhage demonstrates high accuracy and detectability. With its findings of excellent interobserver agreement, our study will be of immense benefit to radiologists.

## Introduction

Susceptibility-weighted imaging (SWI) is a specialized MRI sequence extensively employed in brain examinations. This sequence includes both magnitude and phase images, revealing distinct magnetic susceptibility values in various tissues. SWI serves as an effective tool for investigating tissues with paramagnetic and diamagnetic characteristics, such as calcification and blood [1-3]. Calcification, marked by diamagnetic properties and lower magnetic susceptibility than iron, presents as hyperintense signals in the phase image of a right-hand MR system or hyperdensity exceeding 100 Hounsfield units (HU) in CT scans. Conversely, blood, whether in the form of microbleed or gross hemorrhage, exhibits paramagnetic qualities with higher magnetic susceptibility, resulting in a hypointense signal in the phase image of a right-hand MR system or hyperdensity ranging from 60 to 90 HU in CT scans. Therefore, precise differentiation of these features holds significant value in facilitating the diagnosis of various conditions such as brain tumors, neurological disorders, and traumatic injuries [4-9].

Brain tumors exhibit distinct features regarding various types of intratumoral components including calcification, microbleed, and gross hemorrhage. In 2012, Zulfiqar et al. highlighted that the SWI sequence is highly sensitive in detecting calcification in oligodendrogliomas, with a sensitivity of up to 86% [10]. On the other hand, brain tumors with microbleeds, such as vestibular schwannoma, or those with gross hemorrhage, such as glioblastoma [11,12], also exhibit features detectable by SWI sequence. Therefore, the use of the SWI sequence plays an important role in the accurate differential diagnosis of brain tumors. The use of venous structures as a criterion to distinguish between calcification and bleeding is usually employed [13,14].

Based on our review of research concerning the SWI sequence, in phase images, cortical veins or venous sinuses in the vicinity are commonly used to compare characteristics to distinguish microbleed or gross hemorrhage in the brain from calcification. Cortical veins have the advantage of being distributed throughout the brain, making it easy to compare image characteristics. However, the proximity to cortical arteries poses a risk of potential confusion [15]. In brain tumor cases, a possibility exists that the tumor may compress the nearby cortical veins, making it challenging to observe blood vessel characteristics clearly. Venous sinuses, particularly the superior sagittal sinus (SSS), are frequently employed for comparison due to their substantial size and central location within the brain. Nevertheless, the chance of obtaining phase images differing from normal, due to slow blood circulation, may potentially result in a heterogeneous hypo-hyperintense signal, leading to confusion and difficulties in interpretation [11,13,16].

In light of this, we explored the use of other venous structures for comparison, specifically the internal cerebral vein (ICV). The ICV, being a de-oxygenated blood vessel with paramagnetic properties, provides characteristics similar to microbleeds. Being of large size and centrally located in the brain, it would be easily identifiable without the presence of nearby arteries, thereby reducing confusion. Additionally, no research has been conducted so far using the ICV to distinguish among calcification, microbleed, and gross hemorrhage within brain tumors. Although CT scans remain the gold standard for detecting calcification and blood, they may lack precision in capturing the characteristics of microbleeds. Therefore, the use of the SWI sequence is crucial due to its high sensitivity in detecting microbleed and gross hemorrhage [17,18].

This study is based on the aforementioned information and looks into differentiating among calcification, microbleed, and gross hemorrhage within brain tumors using phase images in the SWI sequence. This differentiation is instrumental in tailoring distinct treatment approaches. We aimed to assess the accuracy of using signal characteristics from the ICV in the SWI sequence to distinguish among calcification, microbleed, and larger hemorrhage within brain tumors, with CT scans for comparison.

## Materials and methods

Study setting and patient selection

This retrospective study was approved by the Institutional Review Board, Royal Thai Army Medical Department. Because of the retrospective nature of the study, the need for informed consent from patients was waived. We included a total of 192 patients with a diagnosis of pretreatment brain tumors including intra-axial and extra-axial masses undergoing MRI and CT examinations from January 2017 to June 2023. Intratumoral components were subdivided into three groups: calcification, microbleed, and gross hemorrhage. Demographic data of all patients were collected from medical records.

CT/MR imaging protocols and data acquisition

All MRI scans were obtained using 1.5T scanners (Ingenia; Philips Healthcare, Amsterdam, Netherlands) with a standard head coil. The patients were imaged using a routine precontrast brain MRI protocol including axial and sagittal T1WI, axial T2WI, axial SWI, and DWI/ADC sequences. SWI data with magnitude and phase images were acquired using the following parameters: echo time msec/repetition time msec, 15/24; flip angle, 15°; bandwidth, 119 Hz per pixel and voxel resolution, 0.5 × 0.5 × 0.5 mm^3^. Routine MRI protocols with gadolinium (Gd) enhancement were obtained in CE-T1WI, CE-3D FLAIR, and CE-3D BrainVIEW (Phillips Healthcare). A standard dose (0.1 mmol/gg) of Gd-DTPA was injected at 1.8 to 2.0 mL/s in all patients using a standard-length IV tubing.

Noncontrast brain CT (NECT) scans were obtained using 640-slice and 128-slice CT scanners (Aquilion One Prism; Canon Medical Systems, Otawara, Japan) with 3.0-mm slice thickness and multiplanar reconstruction. The interval period between MR and CT imaging was within two months.

Imaging and preprocessing analysis

Axial SWI with magnitude and phase images, CE-3D BrainVIEW, and NECT were acquired and evaluated in all patients. All CT and MR imaging findings were independently reviewed by one neuroradiologist with five years of experience in neuroimaging and a general radiologist with 12 years of working experience. The radiologists were blinded to patient data.

**Figure 1:**
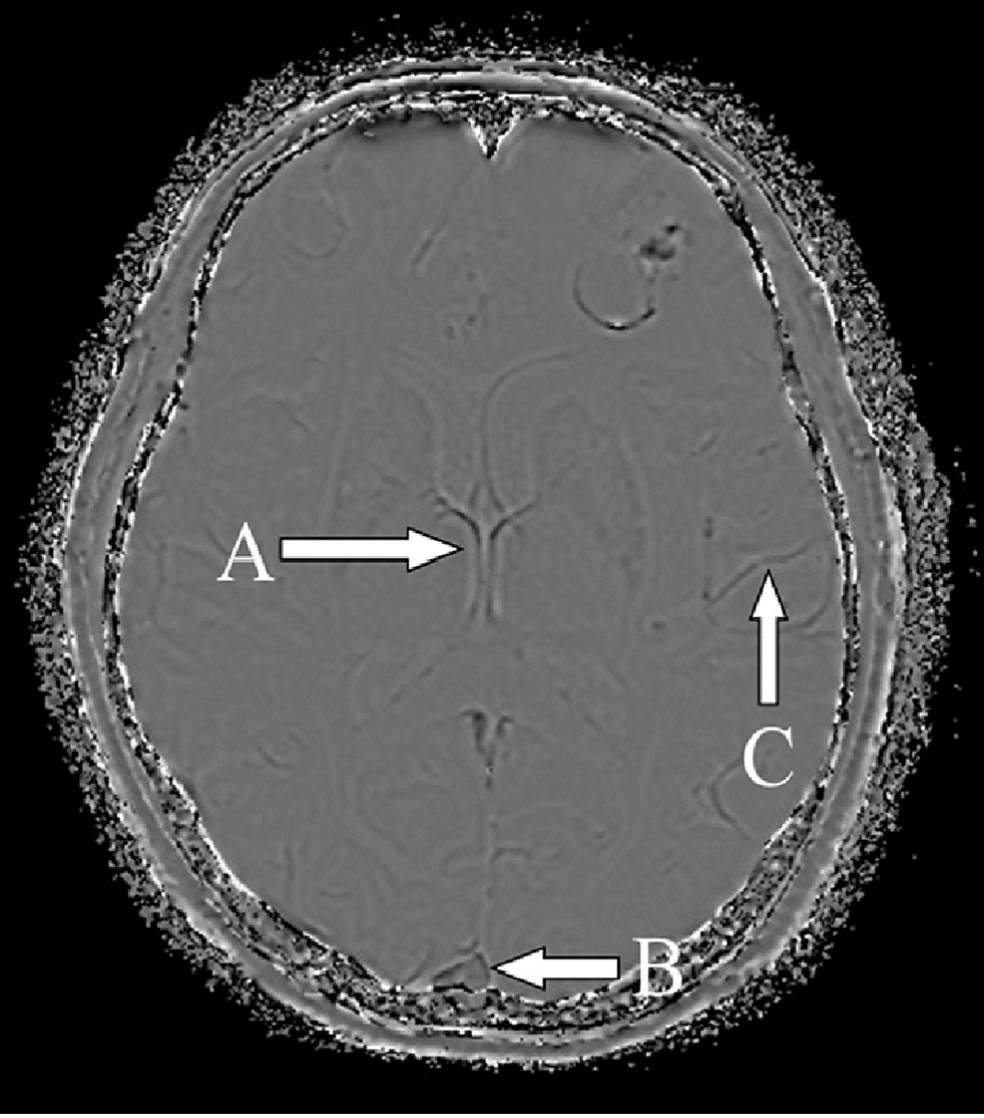
Reference venous structures on SWI phase image including ICV (A), SSS (B), and cortical vein (C) ICV is located in the central image, large in size, and easy to identify ICV: internal cerebral vein; SSS: superior sagittal sinus; SWI: susceptibility-weighted imaging

**Figure 2:**
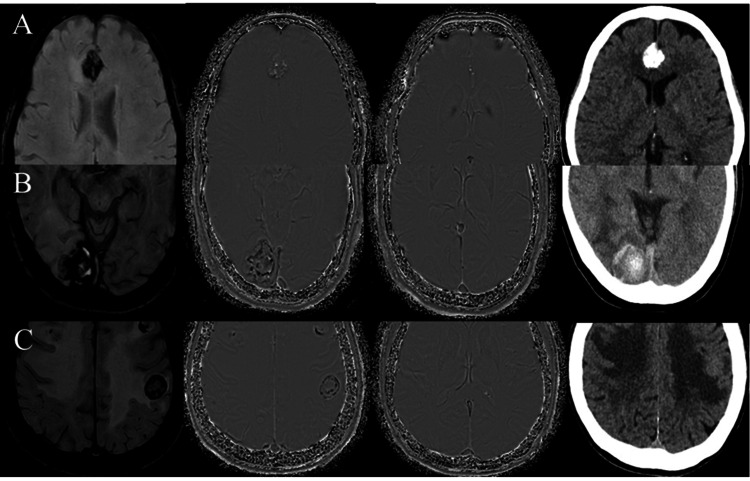
ICVs as the reference venous structure on SWI phase image for differentiating intratumoral calcification (A), gross hemorrhage (B), and microbleed (C) compared with CT CV: internal cerebral vein; CT: computed tomography; SWI: susceptibility-weighted imaging

At the outset, we evaluated the MRI findings including intratumoral susceptibility artifact on SWI sequence on magnitude images. Then we defined the characteristics of the intratumoral susceptibility artifact including calcification, microbleed (size ≤1.0 cm), and gross hemorrhage (size >1.0 cm) by comparing with the reference venous structures, namely, cortical veins, the SSS, and ICV on SWI phase images (Figure 1). The signal intensity of microbleed and gross hemorrhage was similar to the reference venous structures on SWI phase images (Figure 2).

CT Scans to Distinguish Between Calcification and Gross Hemorrhage

Calcification appears as hyperdense areas with greater than 100 HU. Hemorrhagic conditions exhibit hyperdensity ranging between 60 and 90 HU, while microbleeds may not be visible on CT scans. When any disagreements in the interpretation of the radiological results arose between the two radiologists, a consultation was sought with a more experienced neuroradiologist with over 10 years of expertise to reach a consensus agreement.

Statistical analysis

All statistical analyses were performed using STATA, Version 15.1 software (StataCorp LLC, College Station, TX). The interobserver agreement in MRI and CT scans was analyzed using k analysis. A chi-square test was used to evaluate the significance of differences in distinguishing among intratumoral calcification, microbleed, and gross hemorrhage using the reference venous structures including cortical veins, SSS, and ICV. A p-value <0.05 was considered statistically significant.

## Results

Patient demographics, tumor characteristics, and interobserver agreement

The study included a total of 192 patients with 192 intracranial tumors including components of calcification, microbleed, and gross hemorrhage. The demographics and tumor characteristics of the cohort are shown in Table 1. The age of the patients ranged from 2 to 94 years (median: 61 years); 87 were male (45.1%) and 106 were female (54.9%). The predominant diagnoses were meningioma (n = 84, 43.5%), followed by brain metastasis (n = 68, 35.2%), high-grade glioma (n = 21, 10.8%), and others (n = 14, 7.3%). The agreement between the two observers was excellent for calcification, microbleed, and gross hemorrhage with a Cohen's Kappa of 1.

**Table 1:** Demographic data

Variables	Values	Total
Age, years, median (range)	61 (2, 94)	192 patients
≤45, n (%)	21 (10.9%)	
>45, n (%)	171 (89.1%)	
Sex, n (%)		192 patients
Male	87 (45.3%)	
Female	105 (54.7%)	
Diagnosis, n (%)		192 patients
Meningioma	83 (43.2%)	
Brain metastasis	68 (35.4%)	
High-grade glioma	21 (10.9%)	
Schwannoma	4 (2.1%)	
CNS lymphoma	4 (2.1%)	
Oligodendroglioma	3 (1.6%)	
Dural metastasis	2 (1.0%)	
Anaplastic ependymoma	1 (0.5%)	
Germ cell tumor	1 (0.5%)	
Medulloblastoma	1 (0.5%)	
Midline glioma	1 (0.5%)	
Perineural spread of malignant salivary gland	1 (0.5%)	
Pituitary macroadenoma	1 (0.5%)	
Scalp cancer	1 (0.5%)	

Accuracy of ICV, SSS, and cortical veins in detecting intratumoral calcification

Regarding the comparison between ICV and SSS in detecting intratumoral calcification, ICV showed high sensitivity (96.8%) with high specificity (100%), positive predictive value (PPV, 100%), negative predictive value (NPV, 98.5%), and accuracy (98.9%). However, SSS revealed slightly lower detectability with high sensitivity (96.5%), specificity (100%), PPV (100%), NPV (98.3%), and accuracy (98.8%) (p>0.05) (Table 2).

**Table 2:** Accuracy of internal cerebral vein and superior sagittal sinus in detecting intratumoral calcification

	Internal cerebral vein	Superior sagittal sinus	P-value
Sensitivity	96.8 (89.0-99.6)	96.5 (87.9-99.6)	0.873
Specificity	100 (97.2-100)	100 (96.9-100)	1.000
Positive predictive value	100 (94.1-100)	100 (93.5-100)	1.000
Negative predictive value	98.5 (94.6-99.8)	98.3 (94.1-99.8)	0.878
Accuracy	98.9 (96.3-99.9)	98.8 (95.9-99.9)	0.928

As for the accuracy of ICV and cortical veins in detecting intratumoral calcification, as illustrated in Table 3, both showed high sensitivity (96.8 and 96.8%), high specificity (100 and 100%), PPV (100 and 100%), NPV (98.5 and 98.5%), and accuracy (98.9 and 98.9%), respectively (p>0.05). No statistically significant difference was found among ICV, SSS, and cortical veins in detecting intratumoral calcification.

**Table 3:** Accuracy of internal cerebral vein and cortical veins in detecting intratumoral calcification

	Internal cerebral vein	Cortical veins	P-value
Sensitivity	96.8 (89.0-99.6)	96.8 (89.0-99.6)	1.000
Specificity	100 (97.2-100)	100 (97.2-100)	1.000
Positive predictive value	100 (94.1-100)	100 (94.1-100)	1.000
Negative predictive value	98.5 (94.6-99.8)	98.5 (94.6-99.8)	1.000
Accuracy	98.9 (96.3-99.9)	98.9 (96.3-99.9)	1.000

Using the ICV as a reference to characterize calcification, CT revealed intratumoral calcification in 63 of 192 cases. Among these 63 cases, SWI sequence MRI detected calcification in 61 cases, while indicating the absence of calcification in two cases. In two cases, calcification was undetected on MRI but was identified on CT, representing false-negative assessments.

Accuracy of ICV, SSS, and cortical veins in detecting intratumoral microbleed and hemorrhage

A comparative analysis of the intratumoral microbleed/hemorrhage detection between ICV and SSS is shown in Table 4. ICV showed high sensitivity (100%), high specificity (96.8%), PPV (98.5%), NPV (100%), and accuracy (98.9%). However, SSS revealed slightly lower detectability with high sensitivity (100%), specificity (96.5%), PPV (98.3%), NPV (100%), and accuracy (98.8%) (p>0.05).

**Table 4:** Accuracy of internal cerebral vein and superior sagittal sinus in detecting intratumoral microbleed and hemorrhage

	Internal cerebral vein	Superior sagittal sinus	P-value
Sensitivity	100 (97.2-100)	100 (96.9-100)	1.000
Specificity	96.8 (89-99.6)	96.5 (87.9-99.6)	0.873
Positive predictive value	98.5 (94.6-99.8)	98.3 (94.1-99.8)	0.878
Negative predictive value	100 (94.1-100)	100 (93.5-100)	1.000
Accuracy	98.9 (96.3-99.9)	98.8 (95.9-99.9)	0.928

Concerning the accuracy of ICV and cortical veins in the detection of intratumoral microbleed/hemorrhage, as shown in Table 5, both showed high sensitivity (100 and 100%), high specificity (96.8 and 96.8%), PPV (98.5 and 98.5%), NPV (100 and 100%), and accuracy (98.9 and 98.9%), respectively (p>0.05. No significant difference was observed among ICV, SSS, and cortical veins in detecting intratumoral microbleed/hemorrhage.

**Table 5:** Accuracy of internal cerebral vein and cortical veins in detecting intratumoral microbleed and hemorrhage

	Internal cerebral vein	Cortical vein	P-value
Sensitivity	100 (97.2-100)	100 (97.2-100)	1.000
Specificity	96.8 (89-99.6)	96.8 (89.0-99.6)	1.000
Positive predictive value	98.5 (94.6-99.8)	98.5 (94.6-99.8)	1.000
Negative predictive value	100 (94.1-100)	100 (94.1-100)	1.000
Accuracy	98.9 (96.3-99.9)	98.9 (96.3-99.9)	1.000

## Discussion

The presence of intratumoral calcification, microbleed, and hemorrhage plays a crucial role in the differential diagnosis of various tumors. SWI is an excellent tool for identifying these components, with high interobserver agreement observed in our study. The value of using the SWI sequence in detecting calcification and hemorrhagic components has been established in related studies using venous structures as references in phase images, such as SSS or cortical veins. However, no research has been conducted so far as to which venous structure point should be considered for evaluation. Our study indicated high sensitivity, specificity, PPV, and NPV across various levels of comparison, highlighting the effectiveness of using the SWI sequence to characterize intratumoral calcification, microbleed, and hemorrhage. The consistently high accuracy further suggests the reliability of references including the ICV, cortical veins, and SSS.

**Figure 3:**
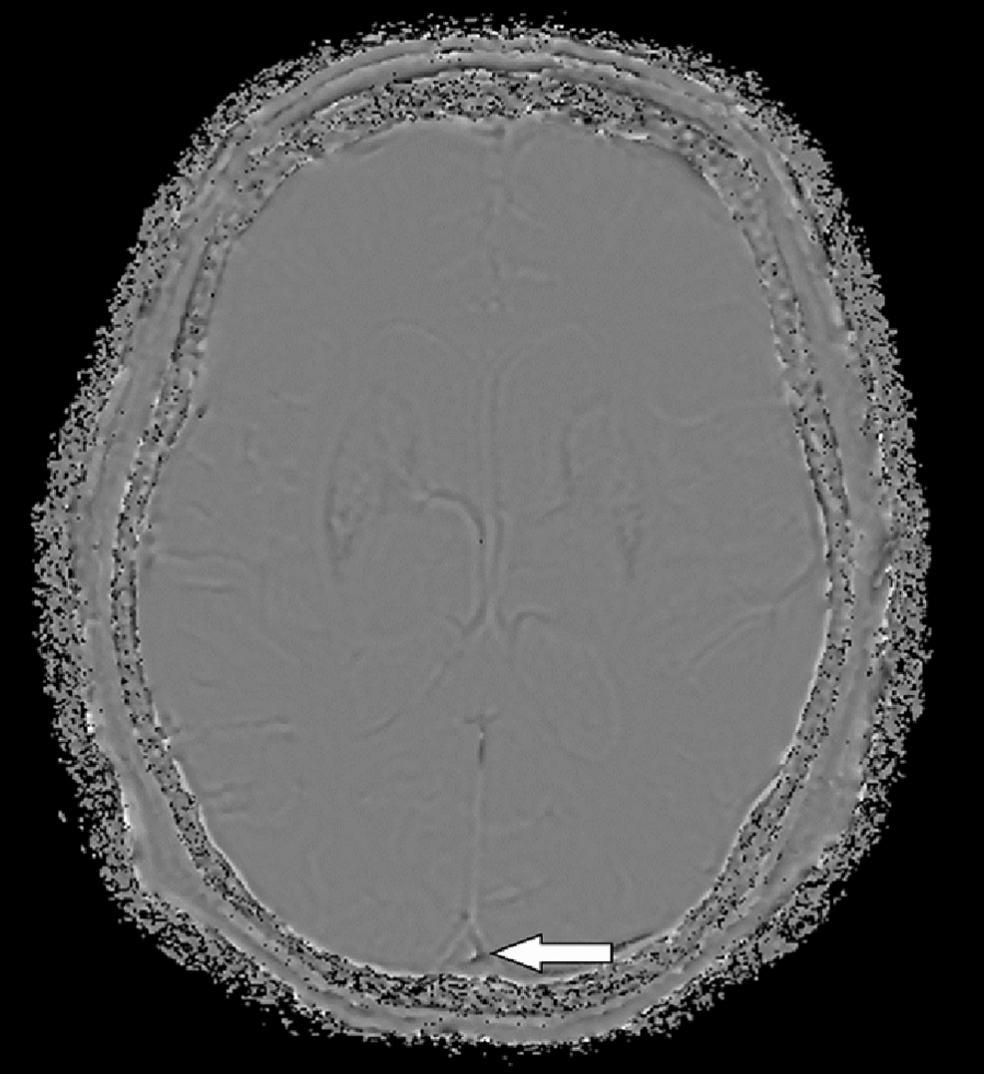
Slow flow in the SSS (white arrow) can misinterpreted as a hyperintense signal of the SWI phase image SSS: superior sagittal sinus; SWI: susceptibility-weighted imaging

As reference points, ICV and cortical veins exhibit no significant differences in distinguishing intratumoral calcification, microbleed, and hemorrhage. They demonstrate equal sensitivity, specificity, PPV, and NPV. In contrast, the SSS shows slightly lower sensitivity, negative predictive value, and accuracy compared with the ICV, albeit without any statistical significance. This discrepancy arose in some cases where signal assessment of the SSS was challenging due to slow flow appearance (Figure 3).

Of note, the positions of the ICV and cortical veins remain unaffected, with the ICV centrally located in the image, and the cortical veins observable from various locations. Despite the challenges in evaluating the SSS, both the SSS and ICV demonstrate no significant difference in distinguishing the intratumoral calcification, microbleed, and hemorrhage.

**Figure 4:**
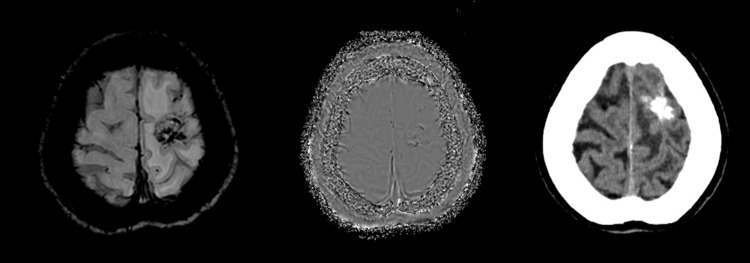
A case of lung cancer with brain metastasis where SWI shows intratumoral microbleed but appears as intratumoral calcification on brain CT This can be due to mixed microbleed and calcified components within brain metastasis CT: computed tomography; SWI: susceptibility-weighted imaging

In addition, SWI detected calcification in 61 cases with a high concordance rate, except for two cases. These particular instances involved brain metastases originating from lung cancer (adenocarcinoma). Notably, brain metastases can exhibit either calcification or hemorrhagic components, and both can coexist within the same lesion. In the first case, a 22-day interval was noted between the CT and MRI scans without any treatment during this period (Figure 4).

In the second case, the imaging revealed an eight-day interval between the CT and MRI scans, and without any recorded history of previous treatment. Therefore, in these two cases, SWI interpreted the findings as microbleed, while CT indicated calcification. These cases may involve both components, resulting in a hypointense signal in the phase image.

Our study has a few limitations, primarily its single-center design. However, this study established the accuracy of using ICV as a reference venous structure on the SWI phase image to characterize intratumoral calcification, microbleed, and hemorrhage. Future research could employ ICV to characterize intratumoral calcification, microbleed, and hemorrhage in nontumoral cases.

## Conclusions

The ICV is located in the central image, is large, without nearby arteries, and is easy to identify on the SWI phase image. Using the ICV as a reference to characterize intratumoral calcification, microbleed, and hemorrhage demonstrates high accuracy and detectability, making it an effective and suitable tool to distinguish these features in brain tumor evaluation. An excellent interobserver agreement was noted, which highlights its benefits in practical use for all radiologists.
